# Prophylaxis of Postoperative Nausea and Vomiting in Adolescent Patients: A Review with Emphasis on Combination of Fixed-Dose Ondansetron and Transdermal Scopolamine

**DOI:** 10.1155/2011/426813

**Published:** 2011-07-02

**Authors:** Joseph V. Pergolizzi, Robert Raffa, Robert Taylor

**Affiliations:** ^1^Department of Medicine, Johns Hopkins University School of Medicine, Baltimore, MD 21205-2196, USA; ^2^Department of Anesthesiology, Georgetown University School of Medicine, Washington, DC 20057, USA; ^3^Department of Pharmaceutical Sciences, Temple University School of Pharmacy, 3307 N Broad Street, Philadelphia, PA 19140, USA; ^4^NEMA Research, Naples, FL 34108, USA

## Abstract

Postoperative nausea and vomiting (PONV) is a relatively common occurrence (20–30%) that delays discharge and, if persistent, can lead to serious complications. The incidence of PONV is a function of patient characteristics, the type and duration of surgery, the type of anesthesia, and the choice of pre-, intra-, and postoperative pharmacotherapy. There are no completely effective antiemetic agents for this condition, but recommendations for treatment strategies are separately available for pediatric and adult patients. Left unclear is whether adolescents should be guided by the pediatric or the adult recommendations. We review the developmental physiology of the relevant physiological factors (absorption, distribution, metabolism, and elimination). We also review the clinical evidence regarding the safety and efficacy of a fixed-dose combination of ondansetron (4 mg, i.v.) and transdermal scopolamine (1.5 mg).

## 1. Epidemiology of PONV in Adult and Pediatric Patients

Postoperative nausea and vomiting (PONV) is a challenging condition with an overall incidence estimated to be around 20% to 30% [1]. With an estimated 46 million inpatient surgical procedures performed in 2006 [2], that amounts to 9 to 13 million cases of inhospital PONV annually. The incidence of PONV may be higher in the burgeoning area of outpatient surgery. One study found that PONV occurs in up to 17% of outpatients in first 48 hours postsurgery compared to only about 10% of inhospital patients who visit a postanesthesia care unit (PACU) [3]. The same investigators determined that PONV delayed patient's discharge and lost revenue for the facility [4].

The occurrence of PONV depends on many factors, including those relating to the patient, for example, age, gender, body weight, history of motion sickness, anxiety, gastroparesis, the underlying disease, type of surgery, and anesthesia. Age has two aspects: PONV is more frequent in pediatric patients with increasing age, that is, over the age of three, but PONV is less frequent in adults with advancing age [1, 5]. Persistent nausea and vomiting may result in dehydration and electrolyte imbalance, and can increase tension on suture lines. Persistent nausea and vomiting has been associated with an increase in venous hypertension and bleeding under skin flaps. In patients with depressed respiration owing to anesthesia or postsurgical analgesics, there is the possibility of inhaling vomit, which could lead to pulmonary complications. PONV is associated with significant morbidity [6], sometimes leading to delayed discharge or hospital readmission. Furthermore, patients find nausea and vomiting highly disagreeable and distressing. In fact, the literature reports that patients regard PONV very negatively [7, 8]. 

The mechanisms behind PONV are the same in adults and children [6]. The physiology of this protective reflex involves several neurotransmitter systems, including dopamine, opioid, histamine, cannabinoid, muscarinic cholinergic, and 5-HT (5-hydroxytryptamine; serotonin receptors). The 5-HT3 subtype of the serotonin receptor pathway plays a major role in the modulation of the PONV reflex. Pharmacologic antagonists of the 5-HT3 receptor have antiemetic properties. Ondansetron (9-Methyl-3-[(2-methyl-1*H*-imidazol-1-yl)methyl]-2,3-dihydro-1*H*-carbazol-4(9*H*)-one), a selective high-affinity competitive antagonist of the 5-HT3 receptor, is a commonly used antiemetic for PONV prophylaxis approved for use in children, and makes no specific differentiations in pediatric dosing beyond weight; doses are the same for adults and pediatric patients 40 kg (88 lb) or larger, regardless of age [9]. 

However, pediatric patients experience more complications in the PACU than adults [10]. In the pediatric population, postoperative vomiting (POV) is more often evaluated than PONV, because it is easier to quantify vomiting than nausea among patients with limited verbal skills. Since pediatric studies of very young patients evaluate mainly POV, the incidence of pediatric PONV is likely underestimated. Nevertheless, the incidence of POV/PONV in young patients is generally held to be about double that of the adult population with overall incidences ranging from 8.9% to 42% and surgery-specific incidences that range from 9% to 80% [11, 12].

Among pediatric patients, one study found a lower rate of POV among children less than three years of age (22–40%) when compared to children over the age of three (42–51%) [13]. While increasing age decreases the risk of POV in adults [1, 5], the opposite is true in pediatric patients. POV incidences are lowest below age three and highest in the range of 11 to 14 years [12, 14]. 

Risk assessment tools exist for PONV in adults. Four independent predictors have been identified (female gender, nonsmoker, history of PONV, and postoperative opioids), and the risk of PONV was 20% for one of these predictors, 40% for two, 60% for three, and 80% if all four were present [15]. A limitation to this and other risk assessment models is that they are designed to assess risk among patient groups rather than individuals [16]. 

These risk assessment tools did not necessarily work well for the full spectrum of pediatric patients; for example, smoking is not applicable, gender does not appear to be a risk factor in prepubescent children, and there are distinct differences in children before and after age three with respect to PONV. Thus, pediatric-specific risk factors have been developed [17]. The risk factors for pediatric POV were found to be age over 3 years, strabismus surgery (but not other types of surgical procedures), duration of surgery over 30 minutes, and history of POV/PONV either in the patient or his or her parents or siblings [18]. While there is little literature available on genetic factors involved in pediatric POV, one study found that monozygotic twins are more likely to have similar POV responses than heterozygote twins [19]. With one positive risk factor using this risk assessment tool, the risk of POV is 10%; with two, the risk is 30%; with three, the risk is 55%; when all four risk factors are present, the risk is 70%. Eberhart and colleagues did not find certain conditions to be independent risk factors of *pediatric* POV: the type of anesthesia (local or regional), intraoperative opioids, postoperative opioids, and female gender [18].

One of the drawbacks to these first attempts at pediatric risk assessments for POV/PONV in children is the fact that the pediatric population is not homogeneous. Age itself is an independent risk factor (older pediatric patients are more likely to have POV than those under three). Female gender was not found to be an independent risk factor unless it was paired with age older female pediatric patients (≥11 years old) are more like their adult counterparts in that female gender confers an independent risk factor [18]. Risk factors specifically for the adolescent patient population have not been thoroughly studied. Expert opinion holds that adolescent risk factors would include female gender, nonsmokers, history of PONV and/or motion sickness, or PONV in relatives. Notwithstanding the information on risk stratification, current practice has adapted a more liberal policy of broad antiemetic prophylactic administration to adolescent patients.

## 2. Adult and Pediatric Patients: Current Treatment Options

The Society for Ambulatory Anesthesia Guidelines recommend these drugs for antiemetic prophylaxis in children: dexamethasone, dimenhydrinate, dolasetron, droperidol, granisetron, ondansetron, perphenazine, and tropisetron [20]. Not all of these agents are approved by the FDA for use in pediatric patients. Ondansetron is a 5-HT3 antagonist and is the only such agent approved for POV prophylaxis in pediatric patients under the age of one month [20]. A dosing comparison derived from this evidence, available mainly in the form of systematic reviews and randomized clinical trials in the literature, demonstrates that dosing for larger children in many cases corresponds to adult dose recommendations. See Table 1.

Ondansetron is generally considered to be the first-line antiemetic for pediatric patients because of its favorable side effect profile [11]. The lowest effective dose in pediatric patients was found to be 0.05 mg/kg [21]. Several pediatric ondansetron studies have confirmed its safety and effectiveness in POV prophylaxis [22–27]. For adults, the fixed dose of ondansetron for PONV prophylaxis is 4 mg IV [9]. The same dose is recommended for a pediatric patient weighing 40 kg (88 lb.) or more. The Centers for Disease Control and Prevention published weight-for-age charts for boys and girls, capturing actual data for 2000, showing that for the 50th percentile, both boys and girls exceed the 88-lb cutoff by the age of 12. Data from those charts are summarized in Table 2.

Furthermore, childhood obesity is on the rise, and the age segment with the steepest increase in obesity is the 12- to 19-year-old pediatric population [30]. Compared to data collected from 1976 to 1980, obesity among 12- to 19-year-olds rose from 5.0% to 17.6% in 2003–2006. (Corresponding obesity rates for other age ranges are 12.4% for 2- to 5-year-olds and 17.0% for 6- to 11-year-olds.)

Since the etiology of PONV in adults and children is multifactorial, prophylactic monotherapy has been limited. Recent consensus guidelines for the management of PONV in adults recommend the combining of antiemetic agents in moderate- to high-risk patients in order to effectively avoid PONV [20]. The combination of ondansetron and transdermal scopolamine may confer increased benefits to patients considered at moderate to high risk for PONV.

## 3. Adolescent Patients: Physiological Considerations

Adolescence is defined as the transitional stage in human development from childhood to adulthood, which commences around the age of 10 to 12. Puberty may be the most significant biologic occurrence during adolescence, which involves a surge in hormone production and a cascade of physical changes. Secondary sexual characteristics appear and the child's body develops into an adult body. Menarche, which commonly occurs in USA girls between the ages of 11 and 13, can be influenced by other factors, including how much relative body fat the girl has [31]. The percentage of body fat is lower at age 20 than at both ages 10 and 40 [32]. A paper on pharmacological research in pediatrics found that drug absorption, distribution, metabolism and elimination found that neonates and babies differed from adults but did not draw such distinctions for older children [33]. 

Whereas there are substantial clinical trial data or good practice guidelines for adult and pediatric use of drugs for PONV, fewer data are available for the specific uses of these drugs for PONV in adolescents. In the absence of such data, it would seem reasonable to extrapolate from the information available for pediatric use at one end and adult use at the other end. However, any special physiology or pharmacology that might be associated with adolescence should first be considered.

The relevant question is whether the adolescent would be an “outlier” in the extrapolation between pediatric and adult response to drugs for PONV. This would occur if pharmacologic response is discordant in this age group, that is, if there are dramatic developmental differences in pharmacokinetics or pharmacodynamics. There is no reason to suspect any difference in pharmacodynamics. Potential differences in pharmacokinetics would involve the drugs absorption, distribution, metabolism, and elimination (ADME). The ontogeny of these processes can be found in recent comprehensive reviews.

### 3.1. Absorption

Due to the relatively higher gastric pH in neonates, absorption of acidic and basic compounds is reduced and enhanced, respectively, and this can contribute to serum concentrations of drugs that are either higher or lower in neonates relative to older children and adults. However, differences in the intestinal absorption physiology of neonates (including higher gastric pH, prolonged gastric emptying, irregular motility, and relatively smaller intestinal surface area) that might affect drug absorption characteristics typically do not extend beyond the early months of life. Maturation of the gastrointestinal tract is usually essentially complete well before two years of age. 

For the special case of transdermal absorption, the epidermis is already well developed in the full-term neonate and is similar to adult skin with respect to epidermal and stratum corneum thickness; it is fully keratinized by one week after birth [34–36]. The skin surface of neonates is characteristically neutral or alkaline (pH 6.2–7.5), but declines rapidly within the first month of life to 5.0–5.5, the range of older children and adults [37–39]. 

Transdermal absorption appears to be independent of age. In an investigation of the properties of skins and matured skin equivalents from foreskin keratinocytes isolated from newborns (2–5 d), children (3–11 yr), and adults (17–58 yr), the percutaneous absorption of hydrocortisone through skins and matured skin equivalents did not vary with age [40].

### 3.2. Distribution

The percentage of body fat and water from birth to 40 years of age has been characterized [41]. The total body water and extracellular water remain relatively constant from one to 40 years old. There is a sharp decrease in body fat of boys at puberty (to about 17%), but no similar decrease in body fat of girls during this period (giving rise to an approximately 1.5 times greater percentage body fat during this period). The distribution of lipophilic compounds may be decreased, and a smaller volume of distribution for lipophilic compounds is expected in boys of this age group.

The physiological variables influencing plasma protein binding in infancy and childhood compared to adult values have been reported [42]. Total plasma protein concentrations are approximately 59 g/L, whereas they are approximately 72 g/L in adults. Fetal albumin has lower binding capacity compared to mature plasma albumin, so plasma protein binding of acidic compounds is reduced in neonates. Binding of basic compounds is also reduced in neonates because of lower levels of plasma globulin and *α*1-acid glycoprotein. However, plasma albumin levels, as well as total protein, plasma globulin, free fatty acids, unconjugated bilirubin, blood pH, and *α*1-acid glycoprotein, are essentially at adult levels by one year of age [42–45]. Both ondansetron and scopolamine have relatively low plasma protein binding.

### 3.3. Metabolism

CYP3A7 is a major cytochrome P450 isozyme expressed during the fetal period, but there is a rapid switch to CYP3A4 perinatally. CYP2E1 activity surges within hours after birth, followed closely by the onset of CYP2D6 expression [46, 47]. CYP3A4, CYP2C9, and CYP2C19 activities appear during the first week of life [48, 49]. CYP1A2, the final hepatic CYP to attain significant expression, does so before 6 months postnatal age [50, 51]. 

Less is known about the developmental pattern of phase II metabolism (glucuronidation and other transferase reactions). Morphine clearance reaches adult levels by three years of age [52], suggesting that metabolism (*via *UGT2B7-mediated glucuronidation) is matured by this time. 

In general, the activity of most drug-metabolizing enzymes (both phase I and phase II), which is lower in the neonate, attains adult levels by six to 12 months of age and no later than three years of age [53].

### 3.4. Elimination

Renal blood flow increases with age, reaching adult rates by six months of age. Renal elimination of drugs depends on glomerular filtration rate (GFR), tubular reabsorption, and tubular secretion. In full-term infants, there is a marked increase in GFR in the first two to three days of postnatal life [54, 55]. Adult values for GFR are attained by six to 12 months of age [55, 56]. 

The proximal convoluted tubules in a full-term infant kidney are small in relation to the glomeruli, thus there is decreased tubular transport of compounds. Tubular function matures at a slower rate than does glomerular function but nevertheless attains adult values by three years old.

## 4. Transdermal Scopolamine

Transdermal scopolamine is an anticholinergic agent which is well absorbed percutaneously and is detected in plasma within four hours after patch application behind the ear; peak plasma concentrations occur within 24 hours in adults [57]. It crosses the placenta and blood-brain barrier. Its half-life after patch removal is 9.5 hours, but it's effects may persist after the patch is removed. 

Transdermal scopolamine is extensively metabolized in the body and conjugated with less than 5% of the total dose excreted unchanged in the urine. The absorption of oral medications may be decreased by transdermal scopolamine because of decreased gastric motility and delayed gastric emptying. Transdermal scopolamine should be used with caution with other drugs capable of causing central nervous system (CNS) effects (such as sedatives, tranquilizers, and alcohol) because of the risk of additive CNS effects (e.g., dizziness). Transdermal scopolamine should be used with caution with other drugs that have anticholinergic effects, including other belladonna alkaloids, antihistamines, tricyclic antidepressants, and muscle relaxants.

**Figure 1:**
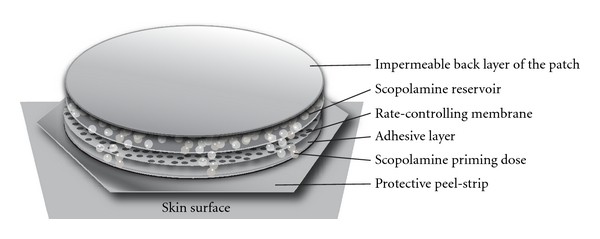
Transdermal scopolamine patch is affixed to skin, where it delivers a priming dose and then a slow release of scopolamine through a special rate-controlling membrane.

The transdermal patch delivery system contains 1.5 mg of scopolamine which is delivered over 72 hours in a continuous slow release through a controlling membrane. The patch is designed so that an initial bolus of scopolamine is administered upon application, followed by continuous release of the drug. See Figure 1.

## 5. Transdermal Scopolamine Studies

A recent randomized, double-blinded study of transdermal scopolamine used as prophylaxis for PONV compared combination therapy (4 mg IV ondansetron plus transdermal scopolamine patch) to ondansetron alone (4 mg IV) in 620 adult females considered at risk for PONV [58]. Patients were undergoing either outpatient laparoscopy or breast augmentation surgery. The study was placebo controlled, in that some patients received a sham patch. Patients were assessed at 24 and 48 hours for PONV. The combination therapy of transdermal scopolamine and ondansetron significantly reduced nausea and vomiting/retching compared to ondansetron alone at 24 hours postsurgery. More patients in the combination group than the ondansetron-only group did not experience vomiting or retching and did not use rescue medication (48% versus 39%, *P* < 0.02). The number of patients who had no nausea, no vomiting/retching, and no rescue medication was also significantly greater in the combination therapy group compared to the ondansetron-only group (35% versus 25%, *P* < 0.01). The combination group had a significantly longer time to first episode of nausea, vomiting/retching, or rescue medication compared to the ondansetron-only group (*P* < 0.05). In addition, the cumulative incidence of adverse events was significantly lower in the transdermal scopolamine plus ondansetron group compared to the ondansetron-only group (36.7% versus 49%,  *P* < 0.01).

## 6. Off-Label Studies of Transdermal Scopolamine in Pediatric Patients

A randomized study of 40 pediatric patients (ages 6 to 14 years) undergoing abdominal surgery under general anesthesia with extradural block received either transdermal scopolamine (140 *μ*g loading dose followed by 5 *μ*g/h) or placebo for the duration of time the child was kept on patient-controlled analgesia (PCA) using morphine [59]. The patients in the transdermal scopolamine group had significantly less incidence of PONV (*P* < 0.001) versus the control group in the first 48 hours after surgery. Side effects in the treated group occurred more often: sedation (*P* < 0.02) and dry mouth (*P* < 0.01) were most commonly reported.

There are no other randomized clinical trials of transdermal scopolamine in the adolescent population. However, there is a study (*n* = 54) of patients aged one to 11 years undergoing strabismus surgery who were randomized to receive a transdermal scopolamine patch or placebo patch for PONV prophylaxis [60]. The rate of PONV was significantly lower in the transdermal scopolamine patch group than placebo (16% versus 48%, *P* < 0.05). Of vomiting episodes that did occur, only single events occurred in the transdermal scopolamine group, while control patients had a median of 3.1 episodes each (range: 1 to 7). No patients in either group complained of any side effects. This particular study reduced dosage in some patients by cutting the patch; this study included no patients over the age of 11.

## 7. Conclusions

The incidence of PONV among adolescent patients is as high as—and likely higher than—the incidence of PONV among adults. However, there are not many FDA-approved treatment options to address adolescent PONV and those that do usually stipulate doses based on body weight typical of patients in the age range of 12 to 17 years (40 kg or 88 lb). Although there are very few off-label studies of transdermal scopolamine in the pediatric population, two have been cited here that found transdermal scopolamine safe and effective [59, 60]. Based on body weight dosing information for ondansetron and the fact that ADME parameters for adolescents are similar to those of adults, it seems reasonable that adolescent patients, ages 12 to 17 years, may derive similar safety and efficacy benefits from the addition of a 1.5 mg transdermal scopolamine patch to a 4 mg IV dose of ondansetron for the prevention of PONV.

## Figures and Tables

**Table 1:** Prophylactic antiemetic agents supported by medical evidence for use in pediatric and adult patients. The high end of the pediatric range often overlaps or is equivalent to the recommended adult dose [20].

Drug	Type of drug	Pediatric dose	Adult dose	Comment
Dexamethasone	Corticosteroid	150 *μ*g/kg up to 5 mg	4-5 mg	Adult and pediatric doses overlap
Dimenhydrinate	Antihistamine	0.5 mg/kg up to 25 mg	1 mg/kg	
Dolasetron	5-HT_3_ antagonist	350 *μ*g/kg up to 12.5 mg	12.5 mg	Adult and pediatric doses overlap
Droperidol	Antidopaminergic drug	10–15 *μ*g/kg up to 1.25 mg	0.625–1.25 mg	Black box warnings, high risk of sedation
Granisetron	5-HT_3_ antagonist	40 *μ*g/kg up to 0.6 mg	0.35–1.5 mg	Adult and pediatric doses overlap
Ondansetron	5-HT_3_ antagonist	50–100 *μ*g/kg up to 4 mg	4 mg	Adult and pediatric doses overlap
Perphenazine	Phenothiazine (antipsychotic)	70 *μ*g/kg up to 5 mg	NA	Only oral formulation is available in USA
Tropisetron	5-HT_3_ antagonist	0.1 mg/kg up to 2 mg	2 mg	Adult and pediatric doses overlap

**Table 2:** Weight for age, 50th percentile, CDC data for 2000 [28, 29].

Age	Boys	Girls
10	72 lb (33 kg)	70 lb (32 kg)
12	92 lb (42 kg)	90 lb (41 kg)
15	136 lb (62 kg)	115 lb (52 kg)
17	142 lb (65 kg)	120 lb (55 kg)
